# Psychosocial work environment and health among ambulance clinicians in northern Sweden

**DOI:** 10.1007/s44192-026-00573-7

**Published:** 2026-08-14

**Authors:** Ann-Therese Hedqvist, Veronica Lindström, Christoffer Ericsson, Jonas Aléx

**Affiliations:** 1 Department of Health and Caring Sciences, Linnaeus University, Kalmar, Sweden; 2 Ambulance Service, Region Kalmar County, Västervik, Sweden; 3 Department of Nursing, Division of Ambulance Service, Umeå University, Region Västerbotten, Umeå, Sweden; 4 School of Business and Healthcare, Arcada University of Applied Sciences, Helsinki, Finland

**Keywords:** Ambulance service, Emergency medical services, Job demands, Occupational health, Prehospital emergency care, Psychosocial work environment, QPS Nordic, Recovery

## Abstract

**Background:**

Ambulance clinicians in emergency medical services (EMS) work under demanding conditions characterised by high workload, unpredictable environments, and exposure to potentially traumatic incidents. These stressors may negatively affect their health and well-being. Understanding how psychosocial work environment factors relate to health outcomes is therefore important for developing preventive strategies in prehospital emergency care. This study aimed to describe ambulance clinicians’ self-rated psychosocial work environment and health in northern Sweden and to examine associations between psychosocial work environment factors and health outcomes.

**Methods:**

An exploratory cross-sectional study was conducted using an anonymous web-based survey of all ambulance clinicians in one northern Swedish region (November 2024; 22 ambulances across eight service areas). Of 187 eligible participants, 134 responded (71.6%). Measures included the General Nordic Questionnaire for Psychological and Social Factors at Work subscales (role clarity, role conflict, job demands, job control, social support, organisational culture, leadership), job satisfaction, self-rated health (SRH), insomnia, exhaustion, anxiety, depression, post-traumatic stress symptoms, and cumulative exposure to traumatic incidents. Analyses included descriptive statistics, Spearman correlations, and group comparisons.

**Results:**

Most ambulance clinicians reported good SRH (83.6%). However, 19.4% screened positive for elevated anxiety, 12.7% for depressive symptoms, 8.2% for clinical insomnia, 6.7% for exhaustion, and 2.2% for probable post-traumatic stress disorder. Higher job demands and role conflict were consistently associated with greater symptom burden and lower SRH, whereas role clarity and job satisfaction showed protective associations. A more positively perceived organisational culture and stronger leadership were also associated with better health outcomes. Clinicians working at urban stations reported higher job demands and role conflict, poorer sleep, higher anxiety and posttraumatic stress, and lower SRH compared with those at rural stations.

**Conclusion:**

Psychosocial work environment factors were closely associated with ambulance clinicians’ health. High job demands and role conflict were consistently linked to poorer health outcomes, whereas role clarity, job satisfaction, supportive leadership, and a positive organisational culture were associated with more favourable health outcomes. These findings highlight organisational factors that may be relevant to consider in efforts to improve workforce health and sustainability in emergency medical services.

**Supplementary Information:**

The online version contains supplementary material available at 10.1007/s44192-026-00573-7.

## Introduction

Ambulance clinicians play a vital role in prehospital emergency care, working under demanding conditions that require rapid decision-making, advanced clinical competence, and close teamwork. Their work is characterised by high operational demands, unpredictable workloads, exposure to potentially traumatic incidents, and complex professional responsibilities [1–5]. These working conditions may affect not only staff well-being but also the quality and sustainability of emergency care delivery [6–8]. Compared with other healthcare professionals, ambulance clinicians report higher levels of work-related stress and burnout [9–11]. Sleep quality and recovery are also recognised as important factors that moderate the effects of stress and workload in shift-based emergency care environments [12–15]. From a mental health perspective, ambulance clinicians represent a high-risk occupational group, where sustained exposure to operational stressors, emotional strain, and disrupted recovery may contribute to the development of common mental disorders.

Although growing attention has been directed toward the psychosocial work environment of ambulance clinicians, important knowledge gaps remain. As highlighted in a recent review [7], much of the existing evidence originates from paramedic-led emergency medical service (EMS) systems, primarily in North America and Australia. In contrast, ambulance services in several European countries, including Sweden, are largely nurse-led and staffed by registered nurses (RNs) or specialist nurses with university-level education. Differences in professional training, organisational structures, and role expectations may shape both working conditions and health outcomes in ways that are not fully captured in the existing literature.

Furthermore, relatively few studies have examined how psychosocial work environment factors relate to health outcomes among ambulance clinicians in Nordic settings [10, 16, 17]. This knowledge gap is particularly relevant given the organisational and geographical characteristics of ambulance services in countries like Sweden, where services often operate across large and sparsely populated regions [18]. Such contextual factors may influence workload, recovery opportunities, and access to support, thereby shaping clinicians’ health and well-being.

Against this background, there is a need for context-specific research that maps the psychosocial work environment and health of ambulance clinicians within nurse-led ambulance systems. The present study therefore aimed to describe ambulance clinicians’ self-rated psychosocial work environment and health in northern Sweden and to examine associations between psychosocial work environment factors and health outcomes.

## Background

The psychosocial work environment is a key determinant of mental health, influencing stress regulation, recovery, and long-term psychological functioning. The work environment encompasses physical, organisational, and psychosocial factors that may influence stress levels, recovery, and long-term health outcomes [19, 20]. A supportive and safe work environment promotes well-being, whereas unfavourable conditions increase the risk of mental distress, burnout, and illness [21–23].

Psychosocial work factors, such as role clarity, job control, interpersonal relationships, and workload, are increasingly recognised as key determinants of occupational health [16, 24, 25]. Deficits in the social and organisational environment are linked to rising rates of mental health problems and sick leave, especially in healthcare professions [26, 27]. In rural healthcare settings, clinicians may face additional challenges such as broader clinical responsibilities, long travel distances, and limited access to specialist support, which may further influence working conditions and well-being [28]. If and how working in rural areas within a nurse-led ambulance service may affect ambulance clinicians’ occupational health is unclear.

Ambulance clinicians are frequently exposed to potentially traumatic incidents, irregular working hours, and high-stakes decision-making, all of which may increase the risk of mental health problems [29–31]. Studies suggest that symptoms of post-traumatic stress disorder (PTSD), depression, anxiety, insomnia, and psychological stress occur more frequently among ambulance clinicians than in the general population [1, 10, 32–34].

Sleep disturbances are common among ambulance clinicians and are closely associated with shift work, irregular working hours, and insufficient recovery between shifts [34–38]. Furthermore, for ambulance clinicians, sleep disturbances, beyond being a consequence of occupational stress, may function as an early indicator of stress-related ill health, with potential downstream effects on fatigue, emotional regulation, and cognitive functioning. Although emotional distancing and cynicism may initially serve as coping strategies in demanding psychosocial work environments, sustained sleep disruption has been associated with exhaustion and other mental health problems among ambulance clinicians [34, 37].

In addition to external stressors, internal and organisational factors—such as professional role conflict (i.e., what is expected of the work versus actual practices), unclear responsibilities, lack of feedback, and perceived mismatches between expectations and actual tasks—also contribute to psychosocial strain [24, 39, 40].

Alongside these demands, ambulance clinicians often describe strong professional identity, meaningfulness, and teamwork as important resources that help them manage the challenges of their work [41–43]. Organisational support, reflective practices, collegial trust, and leadership quality have been identified as key factors that buffer stress and promote resilience [6, 44, 45]. The Job Demands–Resources (JD-R) model provides a useful framework for understanding how demanding work conditions interact with workplace resources to influence health and well-being [6, 46]. Within this perspective, job demands such as workload, emotional strain, and exposure to critical incidents may contribute to strain and burnout, whereas job resources such as autonomy, role clarity, collegial support, and organisational support promote resilience and well-being. In the context of prehospital emergency care, the JD-R model offers a useful perspective for examining how psychosocial work environment factors relate to clinicians’ health. The JD-R model was used as a conceptual lens to contextualise and interpret the selected psychosocial work environment factors and their relationships with health outcomes.

Although studies have demonstrated that both stressors and resources shape the health of ambulance clinicians [4], knowledge about the psychosocial work environment and health among ambulance clinicians in Sweden remains limited. Education and professional roles vary widely across EMS systems globally [47–49]. Understanding indicators of well-being within nurse-led ambulance services is important not only for the services but also for the education of ambulance nurses, as existing evidence on ambulance clinicians’ mental health is primarily derived from paramedic-led organisations. Ambulance services in Sweden operate under differing organisational and geographical conditions, in addition to having differences in professional roles and training. In northern Sweden, ambulance services operate across large geographic areas with long transport distances to hospitals and limited availability of additional emergency medical resources. These contextual conditions may influence workload, recovery opportunities, and access to support, thereby shaping clinicians’ health and well-being.

Despite growing international research on occupational stress in EMS, the need remains for context-specific studies that examine how psychosocial work environment factors relate to health outcomes in nurse-led ambulance systems. Such knowledge may inform organisational strategies to support clinician health and sustainable careers in prehospital emergency care.

## Methods

### Aim

The aim of this study was to describe ambulance clinicians’ self-rated psychosocial work environment and health in northern Sweden and to examine associations between psychosocial work environment factors and health outcomes.

### Design and procedure

This study employed an exploratory, cross-sectional design based on an anonymous web-based survey completed by ambulance clinicians in northern Sweden. The survey included demographic characteristics and established instruments assessing psychosocial work environment factors [50], self-rated health (SRH) [51, 52], mental health symptoms [53–55], PTSD symptoms [56], sleep problems [57], and exhaustion [58–60]. The survey formed part of a broader research programme examining multiple dimensions of work environment, organisational conditions, and health among ambulance clinicians. The present study focuses specifically on psychosocial work environment factors and health outcomes aligned with the study aim.

The study formed part of a larger research project on work environment and health outcomes in prehospital emergency care and was designed to provide a context-specific descriptive overview of psychosocial work conditions and health outcomes within a defined regional ambulance service. The study protocol was preregistered and made publicly available through the Open Science Framework (https://osf.io/wgphu). The study was reported in accordance with the STROBE (Strengthening the Reporting of Observational Studies in Epidemiology) guidelines to ensure transparency, methodological rigor, and reproducibility [61].

### Setting

The study was conducted in a region of northern Sweden as part of a systematic occupational health and safety initiative. The entire regional ambulance service was included, comprising 22 emergency ambulances operating across eight geographic areas and serving a geographically dispersed population. The service includes both urban and rural ambulance stations. Urban stations are located in the region’s larger population centres and are generally characterised by higher call volumes and closer proximity to hospital-based resources, whereas rural stations serve sparsely populated areas with lower call volumes, longer transport distances, and greater geographical dispersion. The service faces ongoing challenges related to recruitment and retention of qualified staff.

Ambulances are staffed by RNs who either hold a bachelor’s degree in nursing or have completed advanced specialist education equivalent to a one-year master’s degree [49, 62]. Nurses work in pairs with either other nurses or emergency medical technicians (EMTs) trained in basic life support and prehospital emergency care. To facilitate clarity in terminology, this study uses the term ‘ambulance clinicians’ to collectively refer to both RNs, specialist nurses, and EMTs working in Sweden’s ambulance service.

### Participants

The study included all ambulance personnel, EMTs, RNs, and specialist nurses employed in direct patient care roles within the regional ambulance service during the data collection period (November 2024). Administrative staff and personnel without clinical responsibilities were excluded. As the study was designed as an exploratory, population-based survey targeting all eligible ambulance clinicians in the region, no a priori sample size or power calculation was performed. Although sample size estimation is recommended for studies that sample from a population to ensure adequate statistical precision and power [63], our primary strategy was to recruit the entire population within a defined region. The effective sample size was therefore determined by the achieved response rate.

### Data collection

Data were collected during November 2024 using a web-based survey administered through the EnterGate platform. Information about the study and a generic survey link were distributed via organisational email and internal communication channels, including the intranet. Because the survey link was generic and not tied to the individual participants, responses were collected anonymously and could not be traced back to specific individuals.

Participation was voluntary, and no incentives were offered. Two reminders were distributed through the same communication channels to support participation and improve the response rate. To reduce response bias, participants were informed that responses would be treated confidentially, reported only at group level, and would not be accessible to their managers in an identifiable form.

The survey included questions on psychosocial work environment factors, health outcomes, and demographic characteristics (see Supplementary Material 1). Because all questionnaire items were mandatory, there were no item-level missing data in the final dataset, and no imputation procedures were required.

### Measures

In the survey, validated instruments were used where applicable, complemented by study-specific measures. Together, these measures capture multiple dimensions of mental health, including affective symptoms, sleep-related disturbances, and stress-related exhaustion among ambulance clinicians.

#### Psychosocial work environment

Selected subscales from the General Nordic Questionnaire for Psychological and Social Factors at Work (QPS Nordic) questionnaire were used to assess psychosocial work environment factors [50]. The included subscales were role clarity (2 items), role conflict (6 items), job demands (16 items), job control (8 items), social support from colleagues (2 items), social support from immediate supervisor (3 items), organisational culture and climate (7 items), and leadership support (7 items). Selected subscales were chosen based on their relevance to the study aim and previous research identifying job demands, role expectations, leadership, and organisational factors as important determinants of health and well-being in emergency care settings. The selection also reduced respondent burden within a comprehensive survey. Responses were rated on a five-point Likert scale ranging from 1 (‘very seldom or never’) to 5 (‘very often or always’). Mean scores were calculated for each subscale, with higher scores indicating higher levels of the measured construct.

Job satisfaction was assessed with three items on enjoyment, satisfaction, and contentment with work [64], rated on a 5-point Likert scale. Higher values indicated greater job satisfaction. Item scores were summed up to create a total score ranging from 3 to 15. The composite score was analysed as both continuous and categorical (low ≤ 7, medium 8–11, high ≥ 12).

#### Health-related outcomes

SRH was assessed using a single, widely used item measuring perceived general health status [51, 52]. Respondents were asked to rate their overall health on a 5-point scale ranging from 1 (very poor) to 5 (very good). This measure is recognised as a robust predictor of morbidity, functional decline, and mortality across different populations [65, 66]. In this study, SRH was analysed as both a continuous variable to capture gradations in perceived health and a dichotomised variable, where responses of 4–5 were categorised as good self-rated health and responses of 1–3 as impaired self-rated health. The dual approach promoted both sensitivity in detecting variation in perceived health and comparability with previous studies that used dichotomised categories.

Sleep problems were evaluated using the 7-item Insomnia Severity Index (ISI) [57]. Questions assessed sleep onset, nighttime awakenings, early awakenings, feeling rested, how sleep issues impacted daily life, and how sleep concerns had affected the individual over the preceding two weeks. Items were rated 0–4, with total scores ranging from 0 to 28. Higher scores indicated more severe problems. Analyses used both the continuous score and a cut-off ≥ 15, based on established guidelines, indicating probable clinical insomnia [57]. Sleep problems were analysed as a separate health outcome due to their established role as both an independent indicator of ill health and a mediator in the relationship between psychosocial stress and mental health outcomes.

Exhaustion was measured using the 6-item version of the Shirom-Melamed Burnout Measure (SMBM-6), which covers physical fatigue, exhaustion, and cognitive weariness [58–60]. Respondents indicated the frequency of symptoms on a 7-point scale (1 = almost never to 7 = almost always). Item scores were summed to create a total score ranging from 6 to 42, with higher scores indicating greater exhaustion. For classification, the total score was divided by six to obtain a mean item score ranging from 1 to 7. A mean item score of ≥ 4.0, equivalent to a total score of ≥ 24, was used to indicate high levels of exhaustion [67]. The shortened version was selected to reduce respondent burden in a comprehensive survey including multiple instruments.

Mental health was assessed using the Hospital Anxiety and Depression Scale (HADS), a validated instrument designed to identify symptoms of anxiety and depression [53–55]. The scale consists of 14 items divided into two subscales: seven measuring anxiety (HADS-A) and seven measuring depression (HADS-D). Responses were given on a 4-point Likert scale (0–3), yielding a possible score range of 0–21 for each subscale. In this study, all seven items of HADS-A were included. One of the seven HADS-D items (‘I can enjoy a good book or radio/TV programme’) was inadvertently omitted during survey construction, resulting in six available depression items and a maximum possible score of 18 for the HADS-D subscale. To identify participants with elevated symptoms, the established cut-off of ≥ 8 was applied for HADS-A [55]. For HADS-D, a proportionally adjusted cut-off of ≥ 7 was used (8/21 × 18 = 6.9, rounded to 7), to approximate the conventional threshold for the full scale. Because this adjusted cut-off has not been formally validated, findings related to depressive symptoms should be interpreted with caution. Subscale scores were analysed both as continuous variables and as dichotomised outcomes.

PTSD symptoms were measured with the Posttraumatic Stress Disorder Checklist for DSM-5 (PCL-5), a validated 20-item self-report measure that reflects the diagnostic criteria for PTSD in the Diagnostic and Statistical Manual of Mental Disorders, Fifth Edition (DSM-5) [68–70]. Participants rated the frequency and severity of symptoms experienced during the preceding 30 days on a scale from 0 (not at all) to 4 (extremely). A total score was calculated, and a cut-off of ≥ 31 was used to indicate symptoms of PTSD, in line with screening recommendations [56, 70].

Exposure to traumatic incidents, defined here as sudden, unexpected clinical incidents that may exceed an individual’s usual capacity to cope and thereby elicit considerable psychological distress [71], was assessed using a 13-item measure covering critical operational incidents (e.g., care of severely injured or dying children, suicide-related callouts, threats or physical violence, feelings of helplessness when no further treatment was possible and unsuccessful resuscitations). The items were developed based on previous EMS research and discussions within the research team, which included experienced ambulance clinicians and researchers. Response options ranged from ‘never’ to ‘yes, many times’. Exposure was coded dichotomously (≥ 1 event) and as a cumulative exposure score, with higher scores indicating more cumulative exposure. The measure was intended to provide an exploratory assessment of cumulative exposure rather than a diagnostic or psychometrically validated trauma scale.

#### Background variables

Demographic variables included gender (male, female or other), age group (20–30 years, 31–40 years, 41–55 years, or ≥ 56 years), years of experience in the ambulance service, professional role (EMT, RN or specialist nurse), primary work location (urban or rural ambulance station), and clinical working time in the ambulance service. Years of experience was categorised into four groups (0–5, 6–10, 11–20, > 20 years) and clinical workload was grouped into three categories: < 75, 75–100, and > 100% of a full-time equivalent (FTE).

#### Scale reliability

Internal consistency of multi-item scales was evaluated using Cronbach’s α, with values ≥ 0.70 considered acceptable [72]. For two-item scales (role clarity), we reported the inter-item correlation and the Spearman-Brown coefficient instead of α.

Cronbach’s α indicated acceptable-to-excellent reliability across measures: job satisfaction (α = 0.87), role conflict (α = 0.77), job demands (α = 0.73), organisational culture/climate (α = 0.83), social support from immediate supervisor (α = 0.82), leadership support (α = 0.87), exposure to traumatic incidents (α = 0.88), PCL-5 (α = 0.95), ISI (α = 0.9), HADS-A (α = 0.86), HADS-D, 6-item (α = 0.82), SMBM-6 (α = 0.92). The job control scale showed slightly lower internal consistency (α = 0.63), which can be expected for short scales. However, the instrument has been validated and widely applied within the Demand–Control–Support model [73] and in subsequent occupational health research, supporting its reliability and construct validity in this context. For the two-item scales, internal consistency was assessed using inter-item correlation and the Spearman-Brown coefficient: role clarity, *r* = .68, r_s_ = 0.81; social support from colleagues, *r* = .62, r_s_ = 0.76. Overall, results support the reliability of the questionnaire constructs among ambulance clinicians in prehospital emergency care. Most instruments used in this study have demonstrated acceptable psychometric properties in occupational and healthcare populations, although several have not been specifically validated in Swedish ambulance clinician populations.

### Statistical analysis

Analyses were conducted in Jamovi version 2.6.26. Descriptive statistics are presented as frequencies and percentages for categorical variables and as means with standard deviations (SDs) or medians with interquartile ranges (IQRs), as appropriate.

Group differences in health outcomes were examined by gender, age, years of experience, occupational role, and primary work location. For two-group comparisons, Welch’s independent-samples t-test or the Mann-Whitney U test was used, depending on data distribution. For comparisons involving more than two groups, Welch’s one-way analysis of variance with Games–Howell post-hoc tests and Kruskal–Wallis analyses with Dunn pairwise comparisons (Holm adjustment) were applied. Effect sizes are reported as Cohen’s d, η^2^, or epsilon-squared (ε^2^), as appropriate. Associations between psychosocial work environment factors and health outcomes were examined using Spearman’s rank correlation (r_s_). All tests were two-sided with a significance level of *p* < .05.

### Ethical considerations

This study is based on anonymised survey data originally collected within the framework of occupational health and safety monitoring in the ambulance service of a region in northern Sweden. According to the Swedish Ethical Review Act (SFS 2003:460), research based on fully anonymous data that does not involve the processing of personal data does not require formal ethical review.

The Swedish Ethical Review Authority issued an advisory opinion confirming that the project fell outside the scope of the Swedish Ethical Review Act (reference number 2025-00945-01). Therefore, formal ethical approval was waived, as the study involved fully anonymised data and posed minimal risk to participants.

Participants were informed that their responses could be used for research purposes and were provided with contact details for the operational manager and lead researcher. Completion of the survey was considered to constitute informed consent. No personal data were processed, and all results are reported at group level.

## Results

We analysed data from 134 ambulance clinicians. Results are presented in this order: (1) participant characteristics; (2) questionnaire data and completeness; (3) prevalence of health outcomes; and (4) associations between psychosocial factors and health, including subgroup comparisons.

### Participant characteristics

Of 187 invited employees, 134 completed the survey (71.6% response rate). Information regarding non-responders was not available due to the anonymous survey design. Consequently, potential non-response bias could not be formally assessed. Data completeness was high; all questionnaire items used in the analyses were fully completed, yielding no missing values. Among participants, 59.7% were male and 39.6% female. The largest age group was 41–55 years (41.0%). Half were specialist nurses (50.0%), and 38.8% had ≤ 5 years’ experience in prehospital emergency care. Most reported a clinical workload of 75–100% FTE (85.8%), and 79.1% worked primarily at urban stations (Table 1).

**Table 1:** Participant characteristics (*n* = 134)

Variable	Category	*n* (%)
Gender	Male	80 (59.7)
	Female	53 (39.6)
	Other	1 (0.7)
Age	20–30 years	18 (13.4)
	31–40 years	43 (32.1)
	41–55 years	55 (41.0)
	≥ 56 years	18 (13.4)
Primary clinical role	Emergency medical technician (EMT)	21 (15.7)
	Registered nurse (RN)	46 (34.3)
	Specialist nurse	67 (50.0)
Work experience in ambulance service	0–5 years	52 (38.8)
	6–10 years	26 (19.4)
	11–20 years	32 (23.9)
	> 20 years	24 (17.9)
Clinical workload (FTE)	< 75%	15 (11.2)
	75–100%	115 (85.8)
	> 100%	4 (3.0)
Primary work location	Urban	106 (79.1)
	Rural (sparsely populated)	28 (20.9)

Values are n (%). Percentages may not total 100 due to rounding. FTE = full-time equivalent

### Health outcomes

**Fig. 1:**
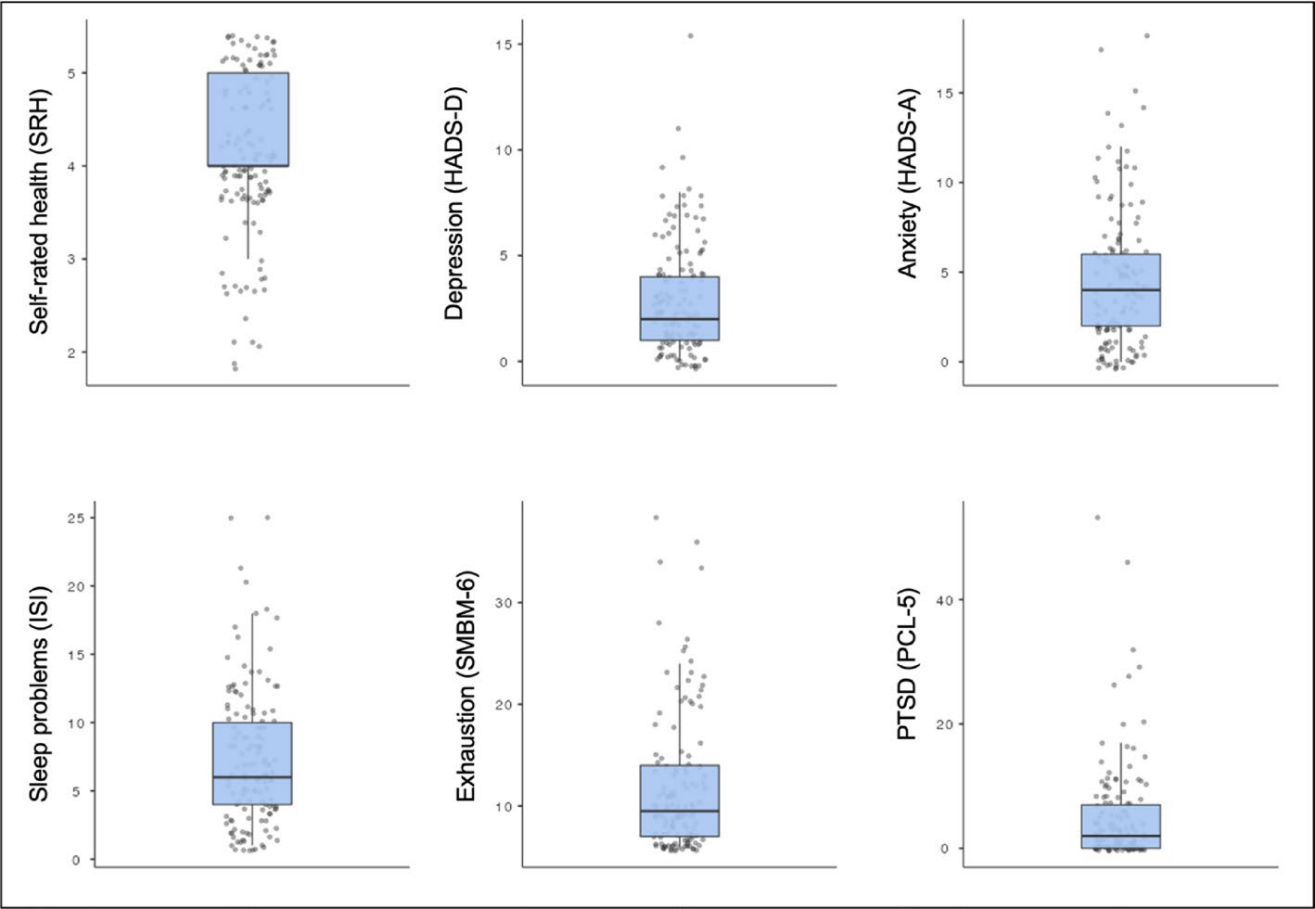
Box plots and scatterplots of health outcomes (*n* = 134): SRH (1–5), HADS-D (0–18), HADS-A (0–21), ISI (0–28), SMBM-6 total score (6–42), PCL-5 (0–80).* Notes* Boxes show medians and interquartile ranges (IQRs); whiskers = 1.5×IQR; dots = individuals. For SRH, higher values indicate better health, whereas the relationship is the inverse for all other variables

Most ambulance clinicians reported good self-rated health (SRH ≥ 4: 83.6%), although 16.4% reported impaired self-rated health. Mean SRH was 4.15 (SD 0.80, range 1–5), indicating generally favourable perceptions of overall health. Regarding specific symptom screens, 8.2% met the threshold for clinical insomnia (ISI ≥ 15), 6.7% for exhaustion (SMBM-6 mean ≥ 4.0), 12.7% screened positive for elevated depressive symptoms (HADS-D ≥ 7, 6-item version), 19.4% for elevated anxiety symptoms (HADS-A ≥ 8), and 2.2% for probable PTSD (PCL-5 ≥ 31). Score distributions for health measures are shown in Fig. 1.

Gender differences were small overall. The only significant difference was for SRH, where females were more likely than men to report good health (92.5% vs. 78.8%; χ^2^/Fisher *p* = .015). No statistically significant gender differences were observed for insomnia, exhaustion, depressive symptoms, anxiety symptoms, or probable PTSD; see Table 2 for gender-stratified prevalences and tests.

**Table 2:** Prevalence of health outcomes by gender (n, % within gender)

Outcome with cut-off					Prevalence by gender, based on cut-off			
	Scale	Min–Max	Mean ± SD	Median (IQR)	Male (*n* = 80)	Female (*n* = 53)	Total (*n* = 134)	Difference (χ^2^ / Fisher *p*)
Self-rated health (SRH)	1–5	2–5	4.15 ± 0.80	4 (1)				0.015*
SRH ≥ 4 (good self-rated health)					63 (79%)	49 (93%)	112 (84%)	
SRH ≤ 3					17 (21%)	4 (8%)	22 (16%)	
Insomnia (ISI)	0–28	0–24	6.4 ± 5.08	6 (6)				0.56
ISI ≥ 15 (clinical insomnia)					8 (10%)	3 (6%)	11 (8%)	
ISI < 15					72 (90%)	50 (94%)	125 (93%)	
Exhaustion (SMBM-6)	6–42	6–38	11.9 ± 6.84	9.5 (7)				1
SMBM-6 mean ≥ 4.0 (exhaustion)					5 (6%)	4 (8%)	9 (7%)	
SMBM-6 mean < 4.0					75 (94%)	49 (92%)	125 (93%)	
Depression (HADS-D)	0–18	0–15	2.99 ± 2.68	2 (3)				0.29
HADS-D ≥ 7 (elevated depressive symptoms)					13 (16%)	4 (8%)	17 (13%)	
HADS-D < 7					67 (84%)	49 (93%)	117 (87%)	
Anxiety (HADS-A)	0–21	0–18	4.49 ± 3.94	4 (4)				0.41
HADS-A ≥ 8 (elevated anxiety symptoms)					13 (16%)	13 (25%)	26 (19%)	
HADS-A < 8					67 (84%)	40 (76%)	108 (81%)	
PTSD (PCL-5)	0–80	0–53	5.24 ± 8.37	2 (7)				0.57
PCL-5 ≥ 31 (probable PTSD)					1 (1%)	2 (4%)	3 (2%)	
PCL-5 < 31					79 (99%)	51 (96%)	131 (98%)	

*p < .05. Percentages in the gender columns are within-gender; the Total column shows % of the total sample. To protect anonymity, gender was recorded as *male*, *female*, or *other*. To protect participant confidentiality, the ‘other’ category (*n* = 1) is not shown separately; totals include all participants. Percentages are rounded to the nearest integer. SMBM-6 mean item score ≥ 4.0 is equivalent to total score ≥  24

#### Correlations between health outcomes

Sleep problems (ISI) were positively associated with exhaustion (SMBM-6; r_s_ = 0.50), depression (HADS-D; r_s_ = 0.48), anxiety (HADS-A; r_s_ = 0.51), and PTSD symptoms (PCL-5; r_s_ = 0.34) and inversely associated with SRH (r_s_ = − 0.47); all *p* < .001. Exhaustion correlated strongly with depression (r_s_ = 0.62), anxiety (r_s_ = 0.68), and PTSD symptoms (r_s_ = 0.52), and inversely with SRH (r_s_ = − 0.50); all *p* < .001. Depression and anxiety were highly correlated (r_s_ = 0.60, *p* < .001). PTSD symptoms correlated with depression (r_s_ = 0.42) and anxiety (r_s_ = 0.47), both *p* < .001, and showed a small inverse association with SRH (r_s_ = − 0.25, *p* = .003). Overall, SRH correlated negatively with all symptom measures (|r_s_| ≈ 0.25–0.50).

Taken together, sleep problems and exhaustion showed the strongest co-occurrence with depression and anxiety, whereas PTSD symptoms displayed weaker associations, and self-rated health was consistently inversely related to all indicators of mental ill-being.

### Psychosocial work environment and association with health outcomes

#### Descriptive data on psychosocial work environment factors

Overall, ambulance clinicians reported high job satisfaction and clear role expectations, along with generally high levels of social support and moderately high scores for organisational culture and leadership. Job demands, role conflict, and job control showed greater variability across individuals. Detailed descriptive statistics and distributional characteristics for all psychosocial work environment factors are presented in Table 3.

Although most clinicians reported low role conflict, nearly one in five reported high levels, primarily related to being expected to manage patients or tasks perceived as outside their professional remit or more appropriate for other healthcare professionals.

All ambulance clinicians reported at least one exposure to traumatic incidents. The most frequently reported incidents were feelings of helplessness when nothing more could be done for a patient, suicide-related callouts, care of severely ill or injured children, and threats or violence from patients or bystanders.

**Table 3:** Descriptive statistics of psychosocial work environment factors

Variable	Scale	Min-Max	Mean ± SD	Median (IQR)
Job demands	16–80	25–60	41.0 ± 5.96	40 (7)
Role conflict	6–30	6–25	14.7 ± 3.84	14 (4)
Role clarity	2–10	2–10	8.12 ± 1.71	8 (1)
Job control	8–40	10–32	17.1 ± 3.90	16.5 (6)
Social support—colleagues	2–10	5–10	8.98 ± 1.16	9 (2)
Social support – supervisor	3–15	3–15	11.4 ± 2.59	12 (3.75)
Organisational culture/climate	7–45	13–44	34.0 ± 5.44	34 (7)
Leadership	7–35	8–35	24.4 ± 5.48	26 (7)
Job satisfaction	3–15	7–15	13.5 ± 1.71	14 (3)

#### Associations with health outcomes

Spearman’s rank correlations between psychosocial work environment factors and health outcomes are presented in Table 4. Poorer psychosocial work conditions were consistently associated with worse health outcomes. Higher job demands showed some of the strongest adverse associations and were linked to poorer self-rated health, higher levels of depressive and anxiety symptoms, greater exhaustion, and more pronounced sleep problems.

Role clarity demonstrated a protective pattern, with higher clarity associated with better self-rated health and lower levels of exhaustion, depressive symptoms, anxiety, and sleep problems. In contrast, role conflict showed the inverse pattern and was among the factors most strongly associated with depressive symptoms, exhaustion, and poorer self-rated health.

Job satisfaction was consistently related to better health across several outcomes, whereas job control showed no meaningful associations with the examined health indicators.

Social support from colleagues and immediate supervisors showed smaller and more selective protective associations, primarily in relation to depressive symptoms and exhaustion.

A more positively perceived organisational culture was consistently associated with better health across multiple outcomes, including self-rated health, mental health symptoms, exhaustion, and PTSD symptoms, and to a lesser extent, sleep problems. Leadership support showed a similar pattern.

Exposure to traumatic incidents showed modest associations with poorer self-rated health and higher levels of depressive symptoms, whereas associations with other health outcomes were weak or negligible.

**Table 4:** Correlation matrix of psychosocial work environment factors and health outcomes (Spearman r_s_)

Health outcome	Job satisfaction	Role clarity	Role conflict	Job demand	Social support (colleagues)	Social support (supervisor)	Organisational culture	Leadership	Exposure to traumatic incidents
Self-rated health (SRH)	0.30***	0.37***	− 0.37***	− 0.38***	0.1	0.11	0.32***	0.19*	− 0.19*
Sleep problems (ISI)	− 0.33***	− 0.19*	0.25**	0.33***	− 0.06	− 0.09	− 0.17	− 0.14	0.08
Depression (HADS-D)	− 0.37***	− 0.34***	0.47***	0.51***	− 0.26**	− 0.26**	− 0.35***	− 0.27**	0.27**
Anxiety (HADS-A)	− 0.18*	− 0.29***	0.34***	0.35***	− 0.05	− 0.14	− 0.28***	− 0.20*	0.09
Exhaustion (SMBM-6)	− 0.34***	− 0.44***	0.42***	0.38***	− 0.14	− 0.23**	− 0.31***	− 0.19*	0.22*
PTSD symptoms (PCL-5)	− 0.12	− 0.18*	0.26**	0.22*	− 0.13	− 0.11	− 0.25**	− 0.21*	0.10

Spearman’s rank correlations (r_s_). *p < .05, **p < .01, ***p < .001. Higher scores have the following meanings for the respective variables: job satisfaction = greater satisfaction; role clarity = clearer roles; role conflict = more conflict; job demand = higher demands; social support (colleagues/supervisor) = greater perceived support; organisational culture = more positive organisational culture/climate; leadership = stronger leadership support; exposure to traumatic incidents = more cumulative exposure to traumatic incidents; SRH = better health; ISI = greater sleep problems; HADS-A/D = more symptoms of anxiety/depression; SMBM-6 = greater exhaustion; PCL-5 = more PTSD symptoms

#### Exploratory subgroup profile–clinical insomnia

Because of the central role of sleep in occupational health and the observed associations between insomnia and multiple health outcomes, an exploratory subgroup analysis was conducted to describe the characteristics of clinicians meeting criteria for probable clinical insomnia (ISI ≥ 15). Eleven clinicians (8.2%) met the ISI cut-off, whereas the remaining participants reported no or mild sleep problems (*n* = 123). Given the small size of the insomnia subgroup and non-normal distributions, exploratory between-group differences were examined using non-parametric tests; means are reported for descriptive purposes, whereas statistical inference is based on Kruskal–Wallis tests.

Clinicians meeting the insomnia cut-off reported higher job demands (M = 47.2 vs. 40.4; χ^2^(1) = 9.86, *p* = .002, ε^2^ = 0.074) and greater role conflict (M = 18.6 vs. 14.3; χ^2^(1) = 11.36, *p* < .001, ε^2^ = 0.085), whereas no differences were observed in role clarity or job control.

The insomnia group also reported poorer self-rated health (M = 3.36 vs. 4.22; χ^2^(1) = 8.64, *p* = .003, ε^2^ = 0.065) and substantially higher symptom levels across multiple outcomes, including depressive symptoms (M = 6.55 vs. 2.67), anxiety (M = 9.36 vs. 4.06), exhaustion (SMBM-6; M = 24.0 vs. 10.8), and PTSD symptoms (M = 13.7 vs. 4.48); all *p* ≤ .004 (ε^2^ ≈ 0.06–0.14).

**Fig. 2:**
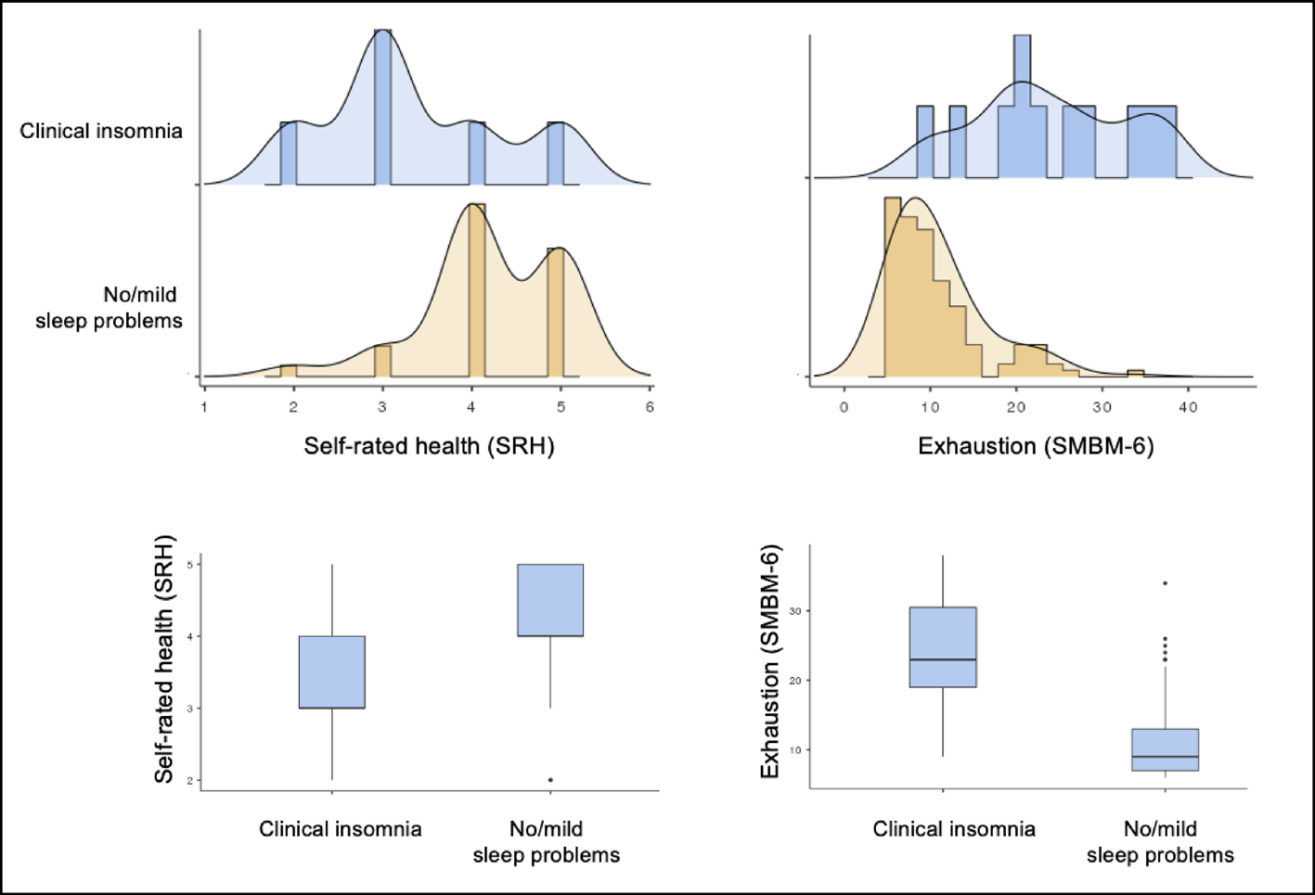
Distributions and between-group contrasts for SRH and exhaustion (histograms/density and boxplots)

In contrast, cumulative exposure to traumatic incidents was lower in the insomnia group than among those without insomnia (M = 23.7 vs. 30.8; χ^2^(1) = 6.45, *p* = .011, ε^2^ = 0.049). Figure 2 illustrates the distributions and between-group contrasts for self-rated health and exhaustion.

Taken together, these exploratory comparisons indicate that insomnia co-occurred with higher current psychosocial strain and broader symptom burden rather than with cumulative exposure to traumatic incidents. These findings are descriptive and should not be interpreted as indicating directional or causal relationships.

#### Group comparisons

Differences in psychosocial work environment and health outcomes were examined based on age, years of experience, professional role, and geographic location of service.

*Age groups.* Across four age groups, anxiety, PTSD symptoms, and cumulative traumatic exposure varied by age (HADS-A: χ^2^(3) = 11.22, *p* = .011, ε^2^ = 0.084; PCL-5: χ^2^(3) = 9.91, *p* = .019, ε^2^ = 0.075; exposure: χ^2^(3) = 14.13, *p* = .003, ε^2^ = 0.106). Post-hoc tests showed lower anxiety in clinicians aged ≥ 56 years versus 20–30 years (*p* = .039), a difference in PTSD symptoms between those aged 41–55 versus 20–30 years (*p* = .014), and more cumulative exposure to traumatic incidents in ambulance clinicians aged ≥ 56 years versus 20–30 or 31–40 years (*p* = .006 and 0.009). No other psychosocial or health outcomes differed by age (all *p* ≥ .10).

*Years of experience.* Across four experience groups (0–5, 6–10, 11–20, and > 20 years), cumulative exposure to traumatic incidents differed significantly by years of experience, χ^2^(3) = 41.4, *p* < .001, ε^2^ = 0.311 (large). Pairwise comparisons showed that clinicians with 0–5 years of experience reported less exposure than those with 6–10 years (*p* = .006), 11–20 years (*p* < .001), or > 20 years of experience (*p* < .001). No significant differences were observed between the three more experienced groups (all *p* ≥ .127). This pattern suggests that exposure to traumatic incidents accumulates early in an ambulance career and thereafter remains relatively stable.

PTSD symptoms also differed across experience groups, χ^2^(3) = 8.63, *p* = .035, ε^2^ = 0.065 (small). Pairwise comparisons indicated higher PTSD symptom scores among clinicians with 11–20 years of experience compared with those with 0–5 years of experience (*p* = .024). No other pairwise differences were observed.

For the remaining outcomes, including job satisfaction, job demand, role clarity, role conflict, job control, self-rated health, sleep problems (ISI), depressive symptoms (HADS-D), anxiety symptoms (HADS-A), and exhaustion (SMBM-6), no statistically significant differences were observed across experience groups (all *p* ≥ .05).

*Primary clinical role.* Across the three roles—EMT, RN, and specialist nurse in prehospital emergency care—only cumulative exposure to traumatic incidents differed significantly, Kruskal–Wallis χ^2^(2) = 6.72, *p* = .035, ε^2^ = 0.050 (small-to-moderate). Dunn–Holm post-hoc tests showed that specialist nurses reported more exposure than RNs (*p* = .029), whereas differences relative to EMTs were not significant (*p* ≥ .25). For other psychosocial and health measures—job satisfaction, job demand, role clarity, role conflict, job control, self-rated health, sleep problems (ISI), depressive symptoms (HADS-D), anxiety (HADS-A), exhaustion (SMBM-6), and PTSD symptoms (PCL-5)—no role-based differences were detected (*p* ≥ .16; ε^2^ ≤ 0.05).

*Primary work location.* Work location was associated with several outcomes. Compared with rural ambulance clinicians, those in urban settings reported higher job demands, more role conflict, more sleep problems, higher anxiety, and more PTSD symptoms, alongside lower role clarity and lower self-rated health (Welch’s ts ≈ 2.3–4.0, all *p* ≤ .028; effects small-to-moderate, |d| ≈ 0.39–0.65). Job control and job satisfaction did not differ by location. Differences for depressive symptoms and exhaustion showed small urban > rural tendencies but were not statistically significant (Welch ps ≥ 0.079). Given the small rural subgroup, estimates for rural clinicians were less precise, and the observed differences between work locations should be interpreted descriptively and with caution.

## Discussion

This study provides a contemporary overview of psychosocial work conditions and health among ambulance clinicians in northern Sweden. Although SRH was generally high, notable proportions reported symptoms of anxiety and depression. Overall, adverse psychosocial conditions, particularly high job demands, and role conflict were associated with poorer mental health and lower SRH. In contrast, role clarity, job satisfaction, supportive leadership, and a positive organisational culture were linked to more favourable health outcomes. Sleep problems were closely intertwined with symptoms of exhaustion, depression, and anxiety, and cumulative exposure to traumatic events increased with years in the service. Differences between urban and rural stations suggest contextual variation in psychosocial strain, with urban settings characterised by higher demands and less favourable health indicators. These findings highlight that health outcomes in ambulance services are shaped not only by exposure to traumatic incidents but also by organisational and contextual work factors. However, the cross-sectional design limits conclusions about causality, and it remains unclear how psychosocial work factors, sleep disturbances, and cumulative trauma exposure interact over time. Future studies should address how psychosocial work conditions, sleep, and exposure to traumatic events jointly influence long-term health outcomes among ambulance clinicians, and whether targeted organisational interventions can reduce these risks.

### Role clarity as a job resource for well-being

The findings suggest a pattern of associations in which high job demands and role conflict were linked to poorer health outcomes, which may be amplified by impaired recovery (e.g., sleep disturbances), thereby increasing the risk of adverse mental health outcomes. The pattern of associations between high job demands, role conflict, and adverse health outcomes aligns with the JD-R framework, which proposes that sustained demands in the absence of adequate resources are associated with psychosocial strain and burnout [6, 46]. In line with previous research, psychosocial work environment factors emerged as central determinants of mental health outcomes in ambulance clinicians [21, 22], reflecting the inherent strain of ambulance work where operational demands often coincide with constrained control [4, 5, 40, 74].

In the present study, role conflict was associated with exhaustion, sleep disturbances, and depressive symptoms, whereas role clarity was consistently associated with more favourable health outcomes. Interpreted through the JD-R framework, these findings suggest that role conflict may represent a demanding aspect of the work environment, whereas role clarity may act as an important job resource that supports health and well-being. These findings highlight the importance of clear remit boundaries and aligned expectations in nurse-led organisational, where professional accountability and clinical responsibility are substantial [4, 24, 75–77]. Although job control is a central construct in established occupational stress models [25, 78], no meaningful associations with the examined health outcomes were observed in this study. This may reflect a true lack of association in this context. However, the lower internal consistency of the subscale may have attenuated the observed relationships, and ambulance clinicians may experience job control differently from the populations in which the scale was originally validated. These findings should therefore be interpreted cautiously. Supportive organisational cultures and strong local leadership were associated with better health outcomes, highlighting their role as important organisational resources within the JD-R framework. This also aligns with evidence that leadership practices, team-based reflection, and feedback structures are critical resources in emergency care settings [6, 44, 45, 79–81]. The elevated demands and poorer health indicators observed at urban stations may indicate that differentiated staffing models, leadership presence, and recovery opportunities warrant further consideration [74, 75]. The lack of differences across age, experience, or professional role underscores that these challenges are systemic, strengthening the case for organisation-level strategies to promote sustainable work environments in nurse-led ambulance services.

### Sleep and recovery as a dual resource

Sleep problems emerged as a central feature of mental health vulnerability in this population. Higher insomnia severity was associated with exhaustion, depressive and anxiety symptoms, PTSD symptoms, and poorer self-rated health, indicating an association between adverse outcomes and impaired sleep. This pattern aligns with growing international evidence showing that sleep disturbances are highly prevalent among ambulance clinicians and closely intertwined with multiple dimensions of mental ill health [34, 37, 38]. From a recovery perspective, insufficient sleep may represent a key pathway through which psychosocial strain translates into downstream health consequences. Viewed using the JD-R framework, where recovery is the mechanism that interrupts the demands-to-strain pathway, insomnia thus functions as the marker of failed recovery. Prior research in EMS has highlighted how irregular working hours, night shifts, and high cognitive and emotional demands constrain opportunities for physiological and psychological recovery, thereby increasing fatigue and reducing resilience over time [74, 82, 83]. Longitudinal evidence from both EMS and broader occupational health research further suggests that persistent sleep disturbances are linked to subsequent exhaustion, depressive symptoms, and reduced work ability, reinforcing the plausibility of recovery pathways underlying these associations [37, 84].

Our findings also resonate with reviews identifying sleep disorders as one of the most consistent factors associated with mental health problems in prehospital emergency medical services [34, 35]. In this context, the observed convergence of insomnia, high job demands, and role conflict may reflect a cumulative load in which organisational pressures erode recovery capacity, rather than isolated risk factors acting independently. Viewed through the JD-R lens, job stress impairs sleep and sleep deprivation leads to fatigue and cognitive impairment, which in turn increases vulnerability to further stress. This creates a vicious cycle, as resource loss begets further resource loss. When job demands increase and persist at very high levels, employees may no longer be able to use adaptive self-regulation strategies and enter a loss spiral of strain and health impairment [85]. This interpretation emphasises recovery as a critical buffer against stress-related ill health in safety-critical, shift-based work [14, 73].

The unexpectedly lower cumulative exposure to traumatic incidents in the insomnia subgroup may partly reflect that cumulative exposure increased with years in service, whereas current sleep problems may be more closely associated with recent psychosocial strain. The exposure measure did not capture the recency or perceived severity of incidents, and selective attrition from the profession or chance due to the small insomnia subgroup cannot be excluded.

From a clinical and organisational standpoint, these results underscore the importance of treating sleep problems as an early warning signal rather than a secondary concern. Interventions that embed recovery into everyday operations, such as fatigue-aware scheduling, limits on consecutive night shifts, protected post-incident recovery time, and access to structured sleep education or stepped-care interventions for insomnia, have been proposed as feasible and potentially high-impact strategies in EMS contexts [38, 86]. In JD-R terms, these are job resources with a dual operating role: they buffer against the impairment pathway, while potentially also supporting the motivational pathway, for instance through work engagement and work ability. Evidence suggests that early-career clinicians and those working in high-demand environments may be particularly vulnerable to deterioration in sleep-related health, highlighting the value of targeted preventive efforts [37, 83]. Future longitudinal/mediation analyses should test whether sleep/recovery mediates the pathway from job demands/role conflict to downstream mental health.

### Exposure to traumatic incidents and correlations with mental health

Cumulative exposure to traumatic incidents increased with years in service, consistent with prehospital emergency care literature describing both the frequency and enduring salience of critical events across an ambulance career [33, 87]. Within a JD-R framework [85], traumatic exposure can be conceptualised as a threat-type demand –acute, integrity-relevant, and qualitatively distinct from chronic hindrance demands such as role conflict. The framework highlights that organisational and interpersonal resources may be important for understanding how different work-related demands relate to health and well-being. In the present study, more exposure was modestly associated with poorer self-rated health and more depressive symptoms, whereas associations with anxiety, sleep problems, exhaustion, and PTSD symptoms were weaker. Although the reasons for this pattern cannot be determined from the present data, previous research suggests that organisational and interpersonal resources may influence how traumatic exposure is experienced and managed over time [44, 45]. The low prevalence of probable PTSD in this sample (2.2%) contrasts with international estimates of 10–20% among ambulance clinicians [10]. Explanations may include true lower burden, symptom expression in other domains (depression or exhaustion) [1, 31, 88, 89], or underreporting and normalisation of distress that suppresses help-seeking [90]. At the same time, consistent with research in prehospital emergency care, repeated exposure to traumatic incidents often co-exists with strong professional identity, collegial solidarity, and a sense of meaning derived from teamwork and patient care [32, 41–43].

Taken together, the findings suggest that both traumatic exposure and organisational stressors are relevant aspects of ambulance clinicians’ work environment. In the present study, organisational stressors such as role conflict are hindrance-type demands, lacking a corresponding occupational buffer, and showed stronger associations with several health outcomes than cumulative exposure to traumatic incidents. This suggests that everyday organisational stressors may represent a more consistent and proximal determinant of mental health than cumulative exposure to critical incidents alone. In practice, trauma-informed supports should be paired with organisational measures that reduce role frictions and workload, routine opportunities for debriefing/reflection and targeted monitoring/support early in careers and in higher-burden urban settings. Ethically, services should counter the normalisation of distress by making psychological health more visible and actionable through systematic screening, timely feedback, and accessible care pathways.

### Clinical implications

From a mental health prevention perspective, the findings identify several organisational factors that may warrant attention. Although the present study cannot determine the effectiveness of specific interventions, the observed associations, together with previous occupational health and EMS research, suggest several areas that may be relevant for future organisational improvement efforts. Psychosocial work environment conditions appear central to both mental health and workforce sustainability among ambulance clinicians. In practice, services should prioritise reducing excessive job demands and role conflict, strengthening role clarity and job satisfaction, and fostering supportive team cultures and first-line leadership. Sleep and recovery require explicit operational safeguards—not as individual coping responsibilities, but as integral components of organisational design. As risk profiles differed by duty location, interventions should be context-sensitive rather than one-size-fits-all. Ethically, organisations have a responsibility to make psychological strain visible and actionable; normalising distress or shifting responsibility to individuals risks preventable harm. Potential organisational considerations informed by both the present findings and previous literature are summarised in Box 1.

**Box 1:** Evidence-informed organisational considerations for supporting workforce health in a nurse-led ambulance service

• *Reduce excessive demands.* Triage low-urgency calls to alternative pathways; add staffing buffers. • *Minimise role conflict.* Clarify remit boundaries; establish inter-service agreements; create rapid feedback loops for recurring boundary issues. • *Strengthen role clarity and job satisfaction.* Structure onboarding, make team debriefs/reflective practice routine, provide regular supervisor feedback, make leadership support visible. • *Build supportive culture and leadership.* Invest in first-line leadership training, participatory problem-solving and a psychosocial safety climate; recognise and reinforce collegial support. • *Support sleep and recovery.* Use fatigue-aware rostering, set limits on consecutive nights, protect recovery windows between missions, provide education on sleep/recovery and targeted follow-up after heavy shifts. • *Monitor leading indicators.* Incorporate brief screens for sleep/exhaustion into routine monitoring to flag emerging risk and trigger timely support.	

### Limitations

Although the study covered the entire regional ambulance workforce and achieved a high response rate (71.6%) with no missing data, the total sample size was relatively small due to the limited population available in this sparsely populated region. This may have reduced statistical power and limits generalisability beyond similar ambulance service contexts. Because information regarding non-responders was unavailable, non-response bias cannot be excluded.

Age and years of experience were analysed as categorical variables to reflect distinct professional stages; however, this categorisation may have reduced variability and limited the ability to examine more fine-grained linear associations with health outcomes.

Another limitation is that the cross-sectional design precludes causal inference; although we observed clear associations between psychosocial work environment factors and health, the temporal ordering of these relationships remains uncertain. All data were self-reported and may be affected by response biases (e.g., social desirability, recall) and common-method variance cannot be ruled out. In addition, the questionnaire did not include measures specifically designed to capture region-specific exposures, such as long transport distances, sparse healthcare infrastructure, or extended on-scene decision-making. As a result, the contextual characteristics of the region could not be empirically examined as distinct stressors and are instead interpreted at a descriptive and contextual level.

Several measurement-related limitations should be considered. Although the included instruments are widely used and generally demonstrate acceptable psychometric properties, most have not been specifically validated in Swedish ambulance clinician populations. The job control subscale showed low internal consistency (α = 0.63), which may have attenuated associations involving this construct. In addition, depressive symptoms were assessed using a modified six-item version of the HADS-D due to the inadvertent omission of one survey item during questionnaire construction, and the traumatic incident exposure measure was not a formally validated instrument. These factors may limit comparability with previous studies and introduce additional measurement uncertainty. Exhaustion was assessed using the shortened SMBM-6 to reduce respondent burden. Although this approach has practical advantages, omission of one dimension of the original SMBQ [91, 92] may have reduced sensitivity to some aspects of exhaustion.

The subgroup analysis of clinicians meeting criteria for probable clinical insomnia should be interpreted with caution due to the small number of participants in this category. The analysis was exploratory and intended to provide descriptive insights rather than support definitive conclusions. Further subgroup analyses were underpowered, limiting precision in those comparisons. Taken together, these limitations warrant cautious interpretation of the findings but do not diminish the value of this region-wide description of psychosocial work conditions and health among ambulance clinicians, which provides an important foundation for future longitudinal and hypothesis-driven research.

## Conclusions

In this cross-sectional survey from a nurse-led ambulance service, psychosocial work environment factors were closely associated with ambulance clinicians’ health. High job demands and role conflict were consistently linked to poorer mental health and lower self-rated health, whereas role clarity, job satisfaction, a positive organisational culture, and stronger first-line leadership were associated with more favourable outcomes. Sleep problems co-occurred strongly with exhaustion, depression, and anxiety, highlighting the importance of recovery, whereas urban duty location emerged as a contextual risk marker.

Although causality cannot be established, the findings identify several organisational factors that may warrant attention in efforts to support workforce health in prehospital emergency care. Together with previous occupational health and EMS research, the results suggest that job demands, role clarity, leadership support, and recovery opportunities may be particularly relevant areas for future intervention and investigation. Longitudinal and multivariable studies are needed to clarify underlying mechanisms and identify key predictors of outcomes such as exhaustion and intention to leave the profession.

## Supplementary Information

Below is the link to the electronic supplementary material.

Supplementary Material 1

## Data Availability

The datasets used and/or analysed during the current study are available from the corresponding author on reasonable request.

## Abbreviations

CI: Confidence intervals
EMS: Emergency medical services
EMT: Emergency medical technician
FTE: Full-time equivalent
HADS: Hospital anxiety and depression scale
HADS-A: Hospital anxiety and depression scale (anxiety)
HADS-D: Hospital anxiety and depression scale (depression)
IQR: Interquartile range
ISI: Insomnia severity index
JD-R: Job demands–resources
PCL-5: Posttraumatic stress disorder checklist for the diagnostic and statistical manual of mental disorders, fifth edition
PTSD: Posttraumatic stress disorder
RN: Registered nurse
SD: Standard deviation
SMBM: Shirom-melamed burnout measure
SRH: Self-rated health
