# Supplementary material for: Psychosocial work environment and health among ambulance clinicians in northern Sweden
Source: Discov Ment Health. 2026 Aug 14;6(1):155. doi: 10.1007/s44192-026-00573-7 (PMC13476205; doi:10.1007/s44192-026-00573-7)
Supplement: Supplementary file 1 [file 44192_2026_573_MOESM1_ESM.docx]

**Survey – English Version**

*This is an English translation of the original Swedish questionnaire used in the study of ambulance clinicians' psychosocial work environment and health. Item wording and response options mirror the Swedish version for measurement equivalence.*

## Introduction

The following survey asks about your work, work environment, health, and well-being. Please answer based on your experiences in the ambulance service.

## Section A. Background – Your Work

1. Gender

- Man
- Woman
- Other
- Prefer not to say

2. Age

- 20–30 years
- 31–40 years
- 41–55 years
- 56 years or older

3. Your primary clinical role

- Emergency medical technician (EMT)
- Registered nurse (RN)
- Specialist nurse

4. Year you completed your current/highest education

- 2024
- 2023
- 2022
- 2021
- 2020
- 2019
- 2018
- 2017
- 2016
- 2015
- 2014
- 2013
- 2012
- 2011
- 2010
- 2009
- 2008
- 2007
- 2006
- 2005
- 2004
- 2003
- 2002
- 2001
- 2000
- 1999
- 1998
- 1997
- 1996
- 1995
- 1994
- 1993
- 1992
- 1991
- 1990
- 1989
- 1988
- 1987
- 1986
- 1985
- 1984
- 1983
- 1982
- 1981
- 1980

5. Years in prehospital emergency care (ambulance service)

- 0–5 years
- 6–10 years
- 11–20 years
- 21–30 years
- 31–40 years
- 41–50 years

6. Clinical workload in ambulance service (percent of full-time equivalent, FTE)

- 15%
- 20%
- 25%
- 30%
- 35%
- 40%
- 45%
- 50%
- 55%
- 60%
- 65%
- 70%
- 75%
- 80%
- 85%
- 90%
- 95%
- 100%
- Over 100%

7. Primary duty location (station/area)

- (Choose)

8. Overall, how satisfied are you with your working hours?

- Very satisfied
- Quite satisfied
- Not very satisfied
- Not at all satisfied

9. Overall, how would you rate the quality of care at your workplace?

- Good
- Fairly good
- Neither good nor poor
- Fairly poor
- Poor

10. How would you rate patient safety at your workplace?

- Good
- Fairly good
- Neither good nor poor
- Fairly poor
- Poor

## Section B. Job Satisfaction

How often do you feel the following about your work?

*Response options: Very often or always / Quite often / Sometimes / Quite rarely / Very rarely or never*

11.1. I enjoy my work.

11.2. I am satisfied with the work I have.

11.3. I feel satisfied with my job.

## Section C. Social and Organizational Work Environment

This section covers psychological safety, job demands/resources, bullying/harassment, role expectations, control, skills/quality, social interactions, leadership, organizational culture, and work–home interference.

### Psychological Safety (Workgroup)

*Response scale: 1 = Does not apply at all … 7 = Applies completely*

12.1. If you make a mistake in your workgroup, you are blamed for it.

12.2. Colleagues in my workgroup sometimes reject others for being different.

12.3. It is difficult to ask members of my workgroup for help.

13.1. Members of my workgroup can raise problems and tough issues.

13.2. It is safe to take interpersonal risks (e.g., voice opinions, admit mistakes, suggest divergent ideas) in my workgroup.

13.3. No one in my workgroup would deliberately undermine my efforts.

14. Anything you would like to add/clarify regarding the items above?

### Job Demands

*Response options: Very rarely or never / Quite rarely / Sometimes / Quite often / Very often or always*

15.1. Do you have to work overtime?

15.2. Do you have to work at a very high pace?

15.3. Do you have too much to do?

15.4. Does your work require quick decisions?

15.5. Are your tasks too difficult for you?

15.6. Does your work require maximum attention?

15.7. Are there disruptive interruptions in your work?

15.8. Are there organizational shortcomings that hinder your work (e.g., unclear directives, outdated guidelines)?

15.9. Does your work require complex decisions?

15.10. Is your work monotonous?

15.11. Do you perform tasks for which you would need more training?

15.12. Does team composition sometimes affect your work negatively?

*Response options: Very rarely or never / Quite rarely / Sometimes / Quite often / Very often or always*

16.1. Are your knowledge and skills useful in your work?

16.2. Does your work involve positive challenges?

16.3. Do you find your work meaningful?

16.4. Does your work require you to acquire new knowledge and skills?

17. Anything you would like to add/clarify regarding the items above?

### Bullying and Harassment (past 12 months)

*Response options: Yes, daily / Yes, weekly / Yes, monthly / Yes, a few times / No*

18. Have you been threatened with violence at your workplace? (Any setting where you perform your work)

19. Have you been exposed to physical violence at your workplace?

20. Have you been subjected to bullying at your workplace? (repeated negative acts over time where you have difficulty defending yourself)

21. Have you experienced offensive conduct via social media, e-mail, or SMS related to your work?

22. Have you been subjected to unwanted sexual attention at your workplace?

19. If you have experienced bullying/harassment, from whom? (Select all that apply)

- Relatives of patients
- Patients
- Colleagues
- Manager
- Prefer not to say

### Role Expectations

*Response options: Very rarely or never / Quite rarely / Sometimes / Quite often / Very often or always*

20. Role clarity

20.1. Are there clearly defined goals for your work?

20.2. Do you know exactly what is expected of you in your work?

21. Role conflict

21.1. Do you have to do things you think should be done differently?

21.2. Are you assigned tasks without being given the resources needed to do them?

21.3. Are incompatible demands placed on you by two or more people?

21.4. Does your work include tasks that conflict with your personal values?

21.5. Do you attend to patients in the ambulance whom you believe belong to another level of care?

21.6. Do you perform tasks that someone else would be more suitable to perform?

22. Anything you would like to add/clarify regarding the items above?

### Control at Work

*Response options: Very rarely or never / Quite rarely / Sometimes / Quite often / Very often or always*

23.1. If there are different ways to do your work, can you choose how to do it?

23.2. Can you influence the amount of work you receive?

23.3. Can you influence decisions about which people you will work with?

23.4. Can you influence decisions that are important for your work?

23.5. Can you set your own work pace?

23.6. Can you decide how long your breaks will be?

23.7. Can you decide when to take a break?

23.8. Can you decide your own working hours?

24. Anything you would like to add/clarify regarding the items above?

### Skill and Quality at Work

*Response options: Very rarely or never / Quite rarely / Sometimes / Quite often / Very often or always*

25.1. Are you satisfied with the quality of the work you do?

25.2. Are you satisfied with your ability to solve problems at work?

25.3. Are you satisfied with your ability to maintain good relationships with coworkers?

25.4. Do you receive feedback on the quality of your work?

25.5. Can you directly judge whether you are doing a good job?

26. Anything you would like to add/clarify regarding the items above?

### Social Interactions

*Response options: Very rarely or never / Quite rarely / Sometimes / Quite often / Very often or always*

27.1. If needed, do you receive support/help in your work from colleagues?

27.2. If needed, do you receive support/help from your immediate supervisor?

27.3. If needed, are your colleagues willing to listen to work-related problems?

27.4. If needed, is your immediate supervisor willing to listen to work-related problems?

27.5. Do you receive appreciation for your work performance from your immediate supervisor?

28. Have you noticed conflicts between coworkers?

*Response options: Very rarely or never / Quite rarely / Sometimes / Quite often / Very often or always*

29. Anything you would like to add/clarify regarding the items above?

### Leadership

*Response options: Very rarely or never / Quite rarely / Sometimes / Quite often / Very often or always*

30.1. Does your immediate supervisor encourage you to take part in important decisions?

30.2. Does your immediate supervisor encourage you to speak up when you disagree?

30.3. Does your immediate supervisor help you develop your skills?

30.4. Does your immediate supervisor address problems as soon as they arise?

30.5. Do you trust top management to secure the future of the workplace/organization?

30.6. Does your immediate supervisor treat staff fairly and equitably?

31. Is the relationship with your immediate supervisor a source of stress for you?

32. Anything you would like to add/clarify regarding the items above?

### Organizational Culture

*Response options: Very rarely or never / Quite rarely / Sometimes / Quite often / Very often or always*

33.1. Are employees at your workplace encouraged to make improvements?

33.2. Is there sufficient communication at your workplace?

33.3. Are people rewarded for a job well done (e.g., pay, recognition)?

33.4. Are employees well cared for at your workplace?

33.5. To what extent is top management interested in staff health and well-being?

34.1. Have you noticed unequal treatment of men and women at your workplace?

34.2. Have you noticed unequal treatment of minorities at your workplace?

34.3. Have you noticed unequal treatment of older vs younger employees at your workplace?

34.4. Are there organizational shortcomings that hinder your work?

35. Anything you would like to add/clarify regarding the items above?

### Work–Home Interface

36. Do demands from your work negatively affect your home/family life?

*Very rarely or never / Quite rarely / Sometimes / Quite often / Very often or always*

37. Do demands from your home/family negatively affect your work?

*Very rarely or never / Quite rarely / Sometimes / Quite often / Very often or always*

### Intention to Leave the Profession

*Response scale: 1 = Strongly agree … 5 = Strongly disagree*

38. I often think about changing profession.

39. I am actively looking for work outside the profession.

40. I would like to leave the profession as soon as possible.

39. If you are considering leaving your current workplace, please describe why.

## Section D. Health

40. How would you rate your general health?

- Good
- Fairly good
- Neither good nor poor
- Fairly poor
- Poor

41. Have you sought care for personal problems or mental health during the past 12 months?

- Yes, in the past 3 months
- Yes, earlier this year but not in the past 3 months
- No

42. Have you sought care for physical problems during the past 12 months?

- Yes, in the past 3 months
- Yes, earlier this year but not in the past 3 months
- No

43. How many times have you been absent from work due to your own illness in the past 12 months?

- None
- 1 time
- 2–3 times
- 4–5 times
- 6 or more times

44. How many times have you gone to work despite feeling you should have stayed at home for health reasons in the past 12 months?

- None
- 1 time
- 2–3 times
- 4–5 times
- 6 or more times

## Section E. Sleep Problems (ISI)

Please answer based on the past two weeks. If you do not have sleep problems, choose the option that best describes your sleep.

45. Sleep difficulties

- Difficulty falling asleep
- Problems waking during the night
- Problems waking too early

*For each: None / Mild / Moderate / Severe / Very severe*

46. How satisfied/dissatisfied are you with your current sleep pattern?

- Very satisfied
- Satisfied
- Neither satisfied nor dissatisfied
- Dissatisfied
- Very dissatisfied

47. To what extent do your sleep problems interfere with daily functioning (e.g., daytime fatigue, work performance, chores, concentration, memory, mood)?

- Not at all
- A little
- Somewhat
- Much
- Very much

48. How noticeable to others is the impairment in your quality of life due to your sleep problem?

- Not at all
- A little
- Somewhat
- Much
- Very much

49. How worried/distressed are you about your current sleep problem?

- Not at all
- A little
- Somewhat
- Much
- Very much

## Section F. Mental Well-being – Part A

For each statement, choose the option that best describes how you have felt during the past week.

50. I feel tense or 'wound up'

- Most of the time
- Often
- From time to time
- Not at all

51. I still enjoy the things I used to enjoy

- Definitely as much
- Not quite so much
- Only a little
- Hardly at all

52. I get a sort of frightened feeling as if something awful is about to happen

- Very definitely and quite badly
- Yes, but not too badly
- A little, but it doesn't worry me
- Not at all

53. I can laugh and see the funny side of things

- As much as I always could
- Not quite so much now
- Definitely not so much now
- Not at all

54. Worrying thoughts go through my mind

- A great deal of the time
- A lot of the time
- From time to time
- Only occasionally

55. I feel cheerful

- Not at all
- Not often
- Sometimes
- Most of the time

56. I can sit at ease and feel relaxed

- Definitely
- Usually
- Not often
- Not at all

57. I feel as if I am slowed down

- Nearly all the time
- Very often
- Sometimes
- Not at all

58. I get a sort of frightened feeling like 'butterflies' in the stomach

- Not at all
- Occasionally
- Quite often
- Very often

59. I have lost interest in my appearance

- Completely
- Very much
- Partly
- Not at all

60. I feel restless as if I have to be on the move

- Very much indeed
- Quite a lot
- Not very much
- Not at all

61. I look forward with enjoyment to things

- As much as I ever did
- Rather less than I used to
- Definitely less than I used to
- Hardly at all

62. I get sudden feelings of panic

- Very often indeed
- Quite often
- Not very often
- Not at all

63. Anything you would like to add/clarify regarding the items above?

## Section G. Mental Well-being – Part B (SMBM‑6)

*Response scale: 1 = Almost never … 7 = Almost always*

64. I feel tired.

65. It feels as if I cannot make it to work in the morning.

66. I feel physically exhausted.

67. I feel I have had enough.

68. My 'batteries' are 'empty'.

69. I feel burned out.

70. My thinking is slow.

71. I have difficulty concentrating.

72. I find it hard to think clearly.

73. I have difficulty focusing my thoughts.

74. I have difficulty thinking about complex matters.

75. I find it difficult to pay attention to my colleagues’ and patients’ needs.

76. I find it difficult to feel emotionally involved with my colleagues and patients.

77. I find it difficult to feel compassion for my colleagues and patients.

65. Anything you would like to add/clarify regarding the items above?

## Section H. Mental Well-being – Part C (PCL‑5)

Answer the following in relation to a stressful event encountered in your ambulance work (e.g., threat of death/serious injury, actual death/serious injury, or sexual violence; directly experienced, witnessed, or learned happened to a colleague). Time frame: past month.

*Response options for each: Not at all / A little bit / Moderately / Quite a bit / Extremely*

66. Repeated, disturbing, and unwanted memories of the stressful experience?

67. Repeated, disturbing dreams of the stressful experience?

68. Suddenly feeling or acting as if the stressful experience were actually happening again (as if you were actually back there reliving it)?

69. Feeling very upset when something reminded you of the stressful experience?

70. Having strong physical reactions when something reminded you of the stressful experience (for example, heart pounding, trouble breathing, sweating)?

71. Avoiding memories, thoughts, or feelings related to the stressful experience?

72. Avoiding external reminders of the stressful experience (for example, people, places, conversations, activities, objects, or situations)?

73. Trouble remembering important parts of the stressful experience?

74. Having strong negative beliefs about yourself, other people, or the world (for example, having thoughts such as: I am bad, there is something seriously wrong with me, no one can be trusted, the world is completely dangerous)?

75. Blaming yourself or someone else for the stressful experience or what happened after it?

76. Having strong negative feelings such as fear, horror, anger, guilt, or shame?

77. Losing interest in activities that you used to enjoy?

78. Feeling distant or cut off from other people?

79. Trouble experiencing positive feelings (for example, being unable to feel happiness or have loving feelings for people close to you)?

80. Irritable behavior, angry outbursts, or acting aggressively?

81. Taking too many risks or doing things that could cause you harm?

82. Being 'superalert' or watchful or on guard?

83. Feeling jumpy or easily startled?

84. Having difficulty concentrating?

85. Trouble falling or staying asleep?

67. Anything you would like to add/clarify regarding the items above?

## Section I. Stressful and/or Traumatic Events in Ambulance Work

A stressful or traumatic event often involves death or risk of death, serious injury, or sexual violence—experienced personally, witnessed, or learned occurred to a colleague.

68. Have you experienced any of the following events in your ambulance work?

*Response options for each: Yes, many times / Yes, a few times / Yes, once / Never*

- Caring for or seeing someone you know with severe injury or illness.
- Caring for or seeing a severely injured or ill child.
- Caring for or seeing a person who attempted suicide or deliberately self-harmed.
- Being exposed to or witnessing violence, threats of violence, or other aggression from a patient or relative.
- Feeling helpless because nothing more could be done to help a patient.
- Feeling uncertain about your responsibility or authority in an acute care situation.
- Receiving incorrect or insufficient information to provide appropriate care in an acute situation.
- Lacking immediate access to a physician or help from colleagues when needed.
- Organizational changes that altered your working conditions.
- A death that affected you emotionally/negatively.
- Difficult or unsuccessful resuscitation.
- A traumatic or tragic event where a patient reminded you of yourself, a family member, or a friend.
- Events involving highly distressed or grieving relatives.

69. Anything you would like to add/clarify regarding the items above?

## Section J. Intrusive Memories / 'Flashbacks'

Intrusive memories are image-based memories that pop into your mind without warning (e.g., a fleeting image or film-like sequence).

70. How often have you experienced intrusive memories of work events…

- … in the past week: Never / Seldom / Sometimes / Quite often / Very often
- … in the past month: Never / Seldom / Sometimes / Quite often / Very often

71. If you have had intrusive memories/'flashbacks', how much have they affected you…

- … in the past week: Not at all / A little / Moderately / A lot
- … in the past month: Not at all / A little / Moderately / A lot

## Final Comments

72. If there is anything else you would like to say about yourself or your work environment, please write it here.

73. If you would like to be interviewed about your psychosocial work environment, please enter your email address (note: only the occupational health service will have access to the combined material with email address).
